# *In vitro *and *in vivo *antitumor effects of acetylshikonin isolated from *Arnebia euchroma (Royle) Johnst *(*Ruanzicao*) cell suspension cultures

**DOI:** 10.1186/1749-8546-4-14

**Published:** 2009-07-11

**Authors:** Wenbi Xiong, Gang Luo, Liming Zhou, Yun Zeng, Wenji Yang

**Affiliations:** 1 Department of Pharmacology, West China Center for Medical Sciences, Sichuan University, Chengdu, Sichuan 610041, PR China

## Abstract

**Background:**

Shikonin derivatives have cytotoxic and antitumor effects. This study aims to investigate the antitumor effects of acetylshikonin isolated from a Chinese medicinal herb *Arnebia euchroma (Royle) Johnst*.

**Methods:**

The 3-(4,5-dimethylthiazol- 2-yl)-2,5-diphenyltetrazolium bromide (MTT) assay was used to determine the *in vitro *antitumor effects of acetylshikonin on human lung adenocarcinoma cell line A549, human hepatocellular carcinoma cell line Bel-7402, human breast adenocarcinoma cell line MCF-7 and mouse Lewis lung carcinoma (LLC) cell line. C_57_BL/6 mice with LLC model were used to study the *in vivo *antitumor effects of acetylshikonin. The expression of bax, bcl-2 and caspase-3 proteins in LLC tissue was determined with immunohistochemical staining.

**Results:**

In A549, Bel-7402, MCF-7 and LLC cell lines, acetylshikonin inhibited cell growth in a dose-dependent manner. IC50 (means ± SD) were 5.6 ± 0.86 μg/ml, 6.82 ± 1.5 μg/ml, 3.04 ± 0.44 μg/ml and 2.72 ± 0.38 μg/ml respectively. Acetylshikonin suppressed tumor growth in C57BL/6 mice with LLC. The inhibition rate of acetylshikonin (2 mg/kg) was 42.85%. Immunohistochemical staining revealed that in the acetylshikonin groups the expression of bax and caspase-3 increased, whereas the expression of bcl-2 decreased, suggesting that acetylshikonin induced tumor cell apoptosis through activating the pro-apoptotic bcl-2 family and caspase-3.

**Conclusion:**

Acetylshikonin isolated from *Arnebia euchroma (Royle) Johnst *cell suspension cultures exhibits specific *in vivo *and *in vitro *antitumor effects.

## Background

**Figure 1:**
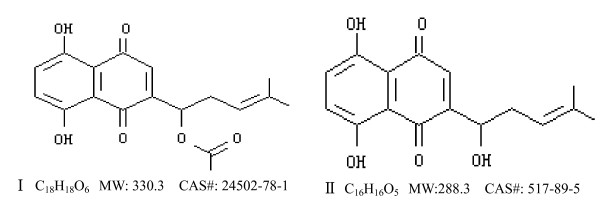
**Chemical structures of acetylshikonin (I) and shikonin (II)**.

*Arnebia euchroma (Royle) Johnst *(*Ruanzicao*), a Chinese medicinal herb that induces apoptosis and exerts antitumor effects, is used to treat inflammatory diseases and cancer [1]. Shikonin derivatives, e.g. shikonin, acetylshikonin (Figure 1), β, β-dimethyl-acrylshikonin, are active components in *Arnebia euchroma (Royle) Johnst*. Natural shikonin-like compounds have *in vitro *inhibitory effects on malignant carcinoma cells. Zhen *et al*. [2] showed that shikonin induced apoptosis of human malignant melanoma A375-S2 cells via activated p53 and caspase-9 pathways. Yoon *et al*. [3] found that shikonin induced HL60 cells apoptosis via caspase-3 dependent pathways. Gao *et al*. [4] reported that shikonin reacted with cellular thiols such as glutathione, and that the depletion of cellular thiols induced apoptosis in HL60 cells. Natural shikonin-like compounds also have significant *in vivo *antitumor effects. In a study by He *et al*. [5], SYUNZ-7, a shikonin derivative, showed antitumor effects both *in vivo *and *in vitro*. Xie *et al*. [6] showed that some shikonin derivatives were more powerful than natural shikonin in terms of antitumor effects on EAC and S180. Kim *et al*. [7] reported that 2-hyim-DMNQ-S33, another shikonin derivative, prolonged the survival time of mice bearing S180.

Due to limited distribution and difficult cultivation of *Arnebia euchroma (Royle) Johnst*, we used the vegetal cell suspension culture technique for the biosynthesis of shikonin-like compounds. Two compounds, namely acetylshikonin and isobutyrylshikonin, have been isolated from the culture vegetal cell suspension.

This study aims to evaluate the *in vivo *and *in vitro *antitumor effects of acetylshikonin extracted from the cell suspension cultures of *Arnebia euchroma (Royle) Johnst*.

## Methods

### Materials

Acetylshikonin extract was obtained from Huakang Pharmaceutical (China) and was confirmed by high-performance liquid chromatography (HPLC). 3-(4,5-dimethylthiazol- 2-yl)-2,5-diphenyltetrazolium bromide (MTT) was obtained from Sigma Chemical (USA). Cyclophosphamide was obtained from Hengrui Pharmaceutical (China).

### Cell lines and cell culture

Malignant cell lines in this study include human lung adenocarcinoma epithelial cell line A549 (ATCC CCL-185), human breast adenocarcinoma cell line MCF-7 (ATCC HTB-22TM) and mouse Lewis lung carcinoma (LLC) (ATCC CRL-1642) were obtained from American Type Culture Collection (USA). Human hepatocellular carcinoma cell line Bel-7402 was obtained from the Cell Bank of the Chinese Academy of Sciences. The cells were cultured in RPMI 1640 (Gibco, USA) supplemented with 10% fetal bovine serum (Minhai Bio-engineering, China) and maintained at 37°C with 4% CO_2 _in a humidified atmosphere. Cell viability was determined with 0.1% trypan blue.

### MTT assay

MTT assay [2] was performed to measure the anti-proliferation effects of acetylshikonin on the cell lines of A549, Bel-7402, MCF-7 and LLC. Acetylshikonin was diluted and added to target cells in triplicates with final concentrations at 25.6, 12.8, 6.4, 3.2, 1.6, 0.8, 0.4 μg/ml. The cells were incubated for 48 hours and 20 μl of 5 mg/ml solution of MTT in phosphate-buffered saline (PBS) was added to triplicate samples and the plates were incubated for additional 4 hours. The plates were then centrifuged and the medium was removed. Two hundred microliters (200 μl) of DMSO was added to each well to dissolve the purple blue sediment, the absorbance was determined at 590 nm on a microplate reader (Model 550, Bio-Rad, USA). The inhibition rate was calculated as follows:



The 50% inhibitory concentrations (IC_50_) of the 48 hours were calculated with Bliss assay.

### Cell growth curve assay

Similarly, A549 cell was used to observe the effects of acetylshikonin on growth curve at various time points. Acetylshikonin was added to A549 cell with various final concentrations (3.2, 1.6, 0.8 μg/ml). MTT assay was performed on the cells in triplicates for each concentration after the cells were incubated for 12, 24, 48, 72 hours respectively. Adriamycin (0.1 μg/ml) served as a positive control.

### Mouse model preparation and treatment

The C_57_BL/6 mice (Experimental Animal Center, West China Center for Medical Sciences, Sichuan University, China) were transplanted with LLC according to protocols of transplanted tumor research. At 24 hours after tumor transplantation, the mice were divided into five groups (12 mice per group) randomly: (1) control group (0.9% normal saline), (2) cyclophosphamide group (60 mg per kg of body weight), (3) acetylshikonin group (0.5 mg per kg of body weight), (4) acetylshikonin group (1 mg per kg of body weight) and (5) acetylshikonin group (2 mg per kg of body weight). Mice in cyclophosphamide group received injections only on Day 1, while mice in all other groups received injections once every two days (six times in total). All injections were carried out intraperitoneally. Data were collected and calculated as follows:

(1) Tumor volume: The length (A) and width (B) of the tumor were measured of the tumor issue of each mouse once every two days since Day 5. Tumor volume (V) = AB^2^/2

(2) Inhibitory rate: The animals were sacrificed on Day 13 and tumors were exercised and weighed. Tumor inhibition rate (%) = (1- tumor tissue weight of treatment group/tumor tissue weight of control group) ×100%

The animal handlings and experimental procedures were approved by the Animal Ethics Committee of Sichuan University.

### Immunohistochemistry

Tumor tissues were fixed with 4% formaldehyde solution at 4°C for 24 hours, dehydrated in graded concentrations of ethanol embedded in paraffin and sliced.

Streptavidin/biotin-peroxidase (SP) method was used for immunohistochemical staining. The primary antibodies, namely bcl-2, bax and caspase-3 (Wuhan Boster Biological Technology, China), were diluted at 1:100. PBS was used as control. Each slice was photographed and the integrated optical density (IOD) was measured with Image pro plus 5.02 (Media Cybernetics, USA).

### Statistical analysis

Data were expressed as mean ± standard deviation (SD) unless otherwise indicated. Statistical differences between the treatment and control groups were determined by Mann-Whitney test with SPSS 12.0 (SPSS, USA). P < 0.05 was considered statistically significant.

## Results

### In vitro effects of acetylshikonin on tumor cell viability

**Figure 2:**
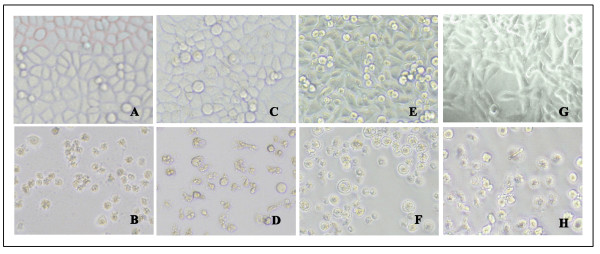
**Acetylshikonin induced morphological changes of tumor cells (×200)**. (A) A549 control; (B) A549 treated with 25.6 μg/ml acetylshikonin; (C) Bel-7402 control; (D) Bel-7402 treated with 25.6 μg/ml acetylshikonin; (E) MCF-7 control; (F) MCF-7 treated with 25.6 μg/ml acetylshikonin; (G) LLC control; (H) LLC treated with 25.6 μg/ml acetylshikonin.

After treatment with acetylshikonin for 48 hours, the untreated tumor cells grew and the cytoskeletons were clearly visible under inverted light microscope. Cells treated with 25.6 μg/ml acetylshikonin became round in shape and condensed nuclei were seen, many of which lost intact membranes, leading to necrosis (Figure 2).

**Figure 3:**
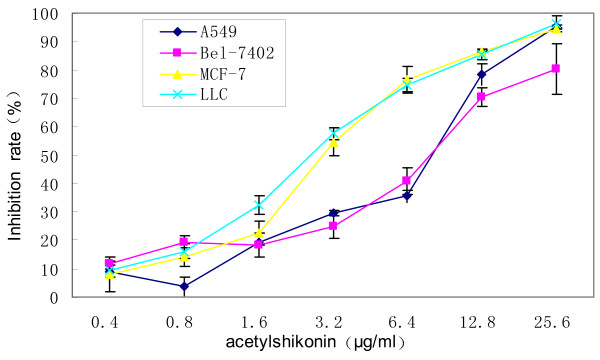
**Inhibitory effects of acetylshikonin on the growth of tumor cells (*n *= 3)**.

**Figure 4:**
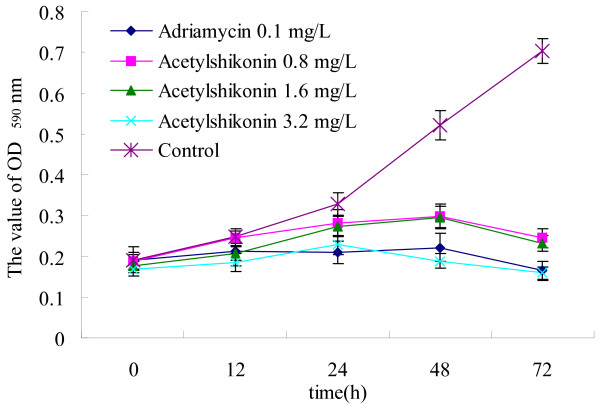
**Inhibitory effects of acetylshikonin on the growth of A549 determined by cell growth curve assay (*n *= 3)**.

Cell inhibition rates were determined by MTT assay. The results revealed that acetylshikonin inhibited the growth of A549, Bel-7402, MCF-7 and LLC in a dose-dependent manner. IC_50 _for 48 hours were 5.6 ± 0.86 μg/ml, 6.82 ± 1.5 μg/ml, 3.04 ± 0.44 μg/ml and 2.72 ± 0.38 μg/ml respectively (Figure 3). Acetylshikonin inhibited the growth of A549 in a time-dependent manner as indicated in the growth curve (Figure 4).

### Effects of acetylshikonin on tumor volume

**Figure 5:**
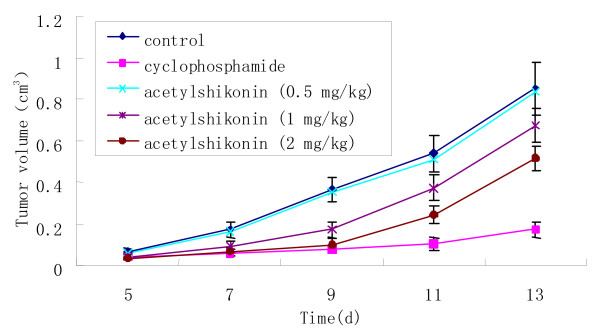
**Inhibitory effects of acetylshikonin and cyclophosphamide on mouse tumor volume**.

Since Day 5, significant differences were observed between the control and cyclophosphamide and acetylshikonin groups. Tumor volume of the mice in the cyclophosphamide group increased slowly, suggesting that the growth of LLC was suppressed by cyclophosphamide. Tumor volume of the mice in the acetylshikonin group (2 mg/kg) was markedly smaller than that in the control group, suggesting that the growth of LLC was suppressed by acetylshikonin (Figure 5).

### Inhibitory rate on LLC

**Figure 6:**
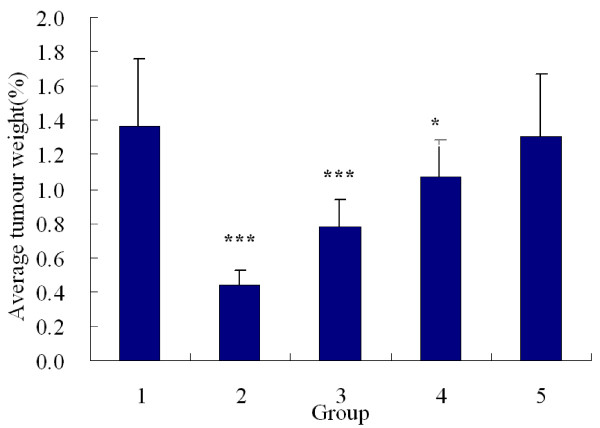
**Effects of acetylshikonin and cyclophosphamide on average tumor weights of LLC in mice (*n *= 12)**. (1) control; (2) cyclophosphamide; (3) acetylshikonin (2 mg/kg); (4) acetylshikonin (1 mg/kg); (5) acetylshikonin (0.5 mg/kg). Acetylshikonin (1 mg/kg, 2 mg/kg) and cyclophosphamide significantly inhibited the growth of LLC. *P < 0.05, ***P < 0.0001, vs. control.

Cyclophosphamide and acetylshikonin (1, 2 mg/kg) had significant inhibitory effects on the growth of LLC in mice. Inhibitory rate of the cyclophosphamide group was 67.81%; inhibition rates of the three acetylshikonin groups were 42.85%, 21.86% and 11.11% respectively, all in a dose-dependent manner (Table 1, Figure 6).

**Table 1:** Inhibitory effects of cyclophosphamide and acetylshikonin on the growth of LLC

			Weight of tumor (g)				
Group (*n *= 12)	Dose (mg/kg)	Inhibition rate (%)	Mean	SD	Percentiles		
					25th	50th (median)	75th
Control	-	-	1.368	0.388	1.070	1.229	1.718
							
Cyclophosphamide	60 × 1	67.81	0.440	0.084	0.371	0.415***	0.523
							
	2 × 6	42.85	0.782	0.154	0.634	0.790***	0.946
Acetylshikonin	1 × 6	21.86	1.067	0.214	0.850	1.010*	1.273
	0.5 × 6	11.11	1.307	0.364	0.962	1.376	1.640

* P < 0.05, ***P < 0.001, vs. control

### Immunohistochemistry evaluation

**Figure 7:**
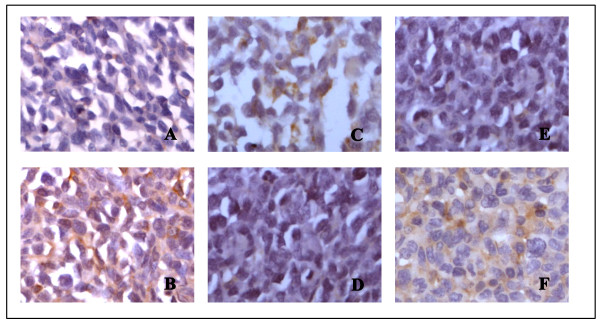
**Bax, bcl-2 and caspase-3 protein expression in LLC tissues (×400)**. (A) Bax control; (B) Bax acetylshikonin (2 mg/kg); (C) Bcl-2 control; (D) Bcl-2 acetylshikonin (2 mg/kg); (E) caspase-3 control; (F) caspase-3 acetylshikonin (2 mg/kg).

The photomicrograph of immunohistochemistry staining is shown in Figure 7. The positive reaction located in cytosol was stained in brown. The color of the stain is positively correlated to the protein expression.

The IOD of each group indicates that the expression of bax and caspase-3 in the acetylshikonin groups increased, whereas bcl-2 decreased in the same groups, resulting in higher bax/bcl-2 ratios, all in a dose-dependent manner (Table 2).

**Table 2:** Bax, bcl-2 and caspase-3 expression (in IOD) in tumor tissues of LLC in mice

				Percentiles		
Group (*n *= 10)	Dose (mg/kg)	Mean	SD	25th	50th (median)	75^th^
**Bax**						
Control	-	13633	2531	11244	13712	15634
Acetylshikonin	2 × 6	48678	2534	46432	48519***	51406
	1 × 6	28502	5064	23628	30232***	33082
	0.5 × 6	21844	4882	16476	22988**	26209
**bcl-2**						
Control	-	19859	2822	17076	20065	22292
Acetylshikonin	2 × 6	8126	1115	7267	7926***	8596
	1 × 6	11171	1459	9916	10814***	12478
	0.5 × 6	15652	1724	14447	15234**	16742
**Bax/bcl-2**						
Control	-	0.70	0.17	0.58	0.74	0.79
Acetylshikonin	2 × 6	6.11	0.92	5.54	6.05***	6.26
	1 × 6	2.56	0.36	2.34	2.47***	2.77
	0.5 × 6	1.41	0.32	1.14	1.29**	1.77
**Caspase-3**						
Control	-	12746	3155	10359	12019	13714
Acetylshikonin	2 × 6	31618	3155	29396	31226***	33530
	1 × 6	22653	4647	18898	21756***	25119
	0.5 × 6	13582	3737	10009	13186	17705

** P < 0.01, *** P < 0.001, vs. control

## Discussion

Recent studies showed that shikonin derivatives acted on multiple tumor cells and triggered multiple cell death pathways. Therefore, shikonin derivatives are potential cancer treatment agents. Acetylshikonin is a main shikonin derivative of *Arnebia euchroma (Royle) Johnst*. Other research on shikonin derivatives also demonstrated that acetylshikonin inhibited K562 and HL-60 tumor growth [8]; however, acetylshikonin had not been studied in detail. The present study indicated that acetylshikonin had *in vitro *and *in vivo *antitumor effects. Acetylshikonin possessed a high level of cytotoxic activity *in vitro*. The present study showed that tumor volume and weight of the mice treated with acetylshikonin increased more slowly than the control *in vivo*.

Apoptosis is critical in the development of tumor. Anti-apoptotic agents such as bcl-2 are initially integral membrane proteins in mitochondria, endoplasmic reticulum (ER) or nuclear membrane [9]. These agents inhibit apoptosis by regulating Ca^2+ ^fluxes through ER membrane [10]. Over expression of bcl-2 turns cells suffering from irreversible gene mutation to normal cell cycle rather than apoptosis, thereby causing cancer. In contrast, bax is a pro-apoptotic molecule that can induce cell apoptosis. In viable cells, bax is either in the cytosol or loosely attached to membranes. In response to a death stimulus, the cytosolic bax translocates to mitochondria where it becomes an integral membrane protein and cross-linkable as homodimers, creating a pathway for cytochrome c to release and activate caspase [11]. As the bcl-2 family proteins act upstream from irreversible cellular damage and all have effects in mitochondria, the ratio of bcl-2 and bax determines whether a cell will live or die [12]. Apoptosis manifests in two major execution programs downstream from the death signal: the caspase pathway and organelle dysfunction, mitochondrial dysfunction in particular. Caspases play an essential role during apoptotic cell death. There are two relatively well characterized caspase cascades: one is initiated by the activation of cell-surface death receptors, such as Fas and tissue necrosis factor, leading to caspase-8 activation which in turn cleaves and activates downstream caspases; the other is triggered by cytochrome c released from mitochondria, which promotes the activation of caspase-9 and caspase-3, thereby initiating caspase cascade to induce cell apoptosis [13].

Our results suggest that acetylshikonin activates the pro-apoptotic bcl-2 family, releases cytochrome c and activates caspase-3, thereby inducing tumor cell apoptosis. Hsu *et al*. [14] found that shikonin activated caspase and induced apoptosis via modulating bcl-2 family, p27 and p53. Liu *et al*. [15] found that certain shikonin derivatives (acetylshikonin) act as modulators of the Nur77-mediated apoptotic pathway and identify a new shikonin-based lead that targets Nur77 for apoptosis induction. Xuan and Hu [8] reported that Shikonin derivatives circumvented diverse cancer drug resistance (P-gp, MRP1, BCRP1, Bcl-2, Bcl-xL) by inducing a dominant necrosis. Apart from inducing apoptosis, acetylshikonin may also inhibit DNA topoisomerase, reduce carcinogenesis and possess antimitogenic and angiogenic actions [16,17]. As a possible wide spectrum agent combating cancer through various mechanisms, acetylshikonin may be a therapeutic candidate.

## Conclusion

Acetylshikonin isolated from *Arnebia euchroma (Royle) Johnst *cell suspension cultures exhibits specific *in vivo *and *in vitro *antitumor effects.

## Abbreviations

A549: human lung adenocarcinoma cell line A549; Bcl-2: B cell lymphoma/lewkmia-2; bax: Bcl-2 associated X protein; Bcrp1: breast cancer resistance protein; Bel-7402: human hepatocellular carcinoma cell line Bel-7402; DMSO: dimethyl sulfoxide; EAC: mice Ehrlich ascitic carcinoma; ER: endoplasmic reticulum; HL60: human promyelocytic leukemia cell line; IC_50_: 50% inhibitory concentrations; IOD: integrated optical density; LLC: Lewis lung carcinoma; MCF-7: human breast adenocarcinoma cell line MCF-7; MRP1: multi-drug resistance protein 1; MTT: 3-(4,5-dimethylthiazol-2-yl)-2,5-diphenyltetrazolium bromide; PBS: phosphate buffered saline; P-gp: P-glycoprotein; S180: C57BL/6 mice fibrosarcoma cell line; SD: standard deviation.

## Competing interests

The authors declare that they have no competing interests.

## Authors' contributions

LMZ and WBX conceived the study design and drafted the manuscript. GL, YZ and WJY conducted the experiments and performed the data analysis. All authors read and approved the final version of the manuscript.
